# Examination of HIV Preexposure Prophylaxis Need, Availability, and Potential Pharmacy Integration in the Southeastern US

**DOI:** 10.1001/jamanetworkopen.2023.26028

**Published:** 2023-07-27

**Authors:** Kristin R. V. Harrington, Christina Chandra, Daniel I. Alohan, Diego Cruz, Henry N. Young, Aaron J. Siegler, Natalie D. Crawford

**Affiliations:** 1 Department of Epidemiology, Rollins School of Public Health, Emory University, Atlanta, Georgia; 2 Department of Medicine, Emory University School of Medicine, Emory University, Atlanta, Georgia; 3 Department of Behavioral, Social, and Health Education Sciences, Rollins School of Public Health, Emory University, Atlanta, Georgia; 4 Department of Clinical and Administrative Pharmacy, University of Georgia College of Pharmacy, Athens

## Abstract

**Importance:**

Daily preexposure prophylaxis (PrEP) use can prevent up to 99% of HIV infections; however, PrEP uptake is low due to poor access to PrEP-prescribing locations for populations at increased risk for HIV, especially in the southeastern US. Pharmacies are a feasible option to increase PrEP access, but little is known about how they could complement current PrEP-prescribing locations.

**Objective:**

To examine geographic distributions of current PrEP-prescribing locations compared with pharmacies and the facility to need ratios (PFNRs) according to HIV risk in the Southeast and describe the potential reach of pharmacies to expand PrEP access.

**Design, Setting, and Participants:**

Data for this cross-sectional study of PrEP-prescribing locations and pharmacies were compiled from January 1 to December 31, 2021. States or specific counties in the Southeast included in this study were jurisdictions identified as high-priority areas for the Ending the HIV Epidemic in the US (EHE) initiative.

**Exposure:**

Expansion of HIV prevention services to pharmacies.

**Main Outcomes and Measures:**

Choropleth maps of 5-year HIV risk per 100 000 persons were developed for EHE jurisdictions in the southeastern US. PrEP-prescribing locations (obtained from a national database of PrEP prescribers) and pharmacies (obtained from state pharmacy boards) were overlayed on HIV risk maps. The PFNRs by state were calculated as number of facilities (PrEP-prescribing locations or pharmacies) divided by 5-year HIV risk per 100 000 persons. Lower PFNRs indicated lower geographic availability of locations to meet the needs of the population at risk for HIV. The PFNRs for current PrEP-prescribing locations vs pharmacies were compared.

**Results:**

Among the 2 southeastern states and 13 counties in 4 southeastern states included, PrEP-prescribing locations were unequally distributed across EHE areas, with substantially fewer in areas at high risk for HIV. Pharmacies were evenly dispersed across areas regardless of HIV risk. The mean PFNR across all states for current PrEP-prescribing locations was 0.008 (median, 0.000 [IQR, 0.000-0.003]); for pharmacies, it was 0.7 (median, 0.3 [IQR, 0.01-0.1]). The PFNRs were at least 20.3 times higher for pharmacies compared with PrEP-prescribing locations. States with the greatest potential increase in PFNRs with expansion to pharmacies included Kentucky, South Carolina, and Tennessee.

**Conclusions and Relevance:**

The findings of this cross-sectional study suggest that expanding HIV prevention services to pharmacies in EHE areas in the Southeast could significantly increase capacity to reach individuals at increased risk of HIV transmission. Legislation aimed at allowing pharmacists to prescribe PrEP and provide HIV prevention services may be an important next step in ending the HIV epidemic.

## Key Points

**Question:**

What is the potential reach of pharmacies in helping to expand preexposure prophylaxis (PrEP) access across the southeastern US?

**Findings:**

In this cross-sectional study of 6 states in the southeastern US, pharmacies had greater ability to meet the need for PrEP among residents of the southeastern US compared with current PrEP-prescribing locations. States that could see the greatest benefit from expansion of PrEP prescribing to pharmacies included Kentucky, South Carolina, and Tennessee.

**Meaning:**

These findings suggest a substantial potential increase in HIV prevention and care services if services were expanded to pharmacies across the southeastern US.

## Teaser

This cross-sectional study evaluates the current burden of HIV in communities across the southeastern US with current preexposure prophylaxis (PrEP)-prescribing locations and pharmacies and calculates the ratio of location to need.

## Introduction

In 2011, the Institute of Medicine’s Committee on HIV Screening and Access to Care called for more medical professionals trained in HIV prevention and care to meet the needs of the growing HIV epidemic.^1^ Since preexposure prophylaxis (PrEP) was approved for use among those at high risk of contracting HIV in 2012, there has been a steady but slow increase in PrEP uptake. In 2015, only 3% of individuals indicated for PrEP had received a prescription, but as of 2020, that number increased to 25%.^2^ Notably, populations at the highest risk of HIV transmission, namely Black men who have sex with men, particularly those who live in the US South, have the lowest PrEP uptake.^3^ About 60% of these men are indicated for PrEP, yet less than 10% in the South received PrEP prescriptions.^4^

While some reasons for low PrEP uptake are related to individual risk perceptions and stigma, structural barriers persist as a key driver of PrEP prescriptions for Black men who have sex with men. For example, many health care facilities are inaccessible to populations at the highest risk of HIV transmission.^5,6^ Moreover, health care facilities often lack the capacity to screen for PrEP, resulting in failures to prescribe PrEP when indicated.^7^ Only about 17% of residency programs in nonrural communities and none in rural communities train their residents on HIV prevention.^8^ These critical access barriers create circumstances in which populations who need HIV prevention services the most cannot access them; and when they can access them, there may be a lack of capacity for clinicians to provide HIV prevention services. This requires public health to identify solutions that could both enhance access to HIV prevention services to reach those at the highest need and ensure adequate training of the medical community to provide these services.

Pharmacies are a viable solution to this critical problem. Pharmacies are, on average, located within about 5 miles of every US household.^9^ The 2022-2025 National HIV/AIDS Strategy for the US calls for the integration of pharmacists into the provision of care for people living with and at risk for HIV to meet the growing needs of the HIV epidemic.^10^ In a cross-sectional survey of accredited pharmacy schools in the US, 89% of respondents included some didactic HIV prevention curriculum in their program.^11^ Moreover, extensive training, both in person and virtually, provides education on expanded HIV prevention services for the pharmacy environment.^12^ However, HIV prevention services are not routinely offered in pharmacies, even in neighborhoods with high baseline HIV levels. This may be due in part to the wide range of HIV prevention services that pharmacies are capable of providing. For example, almost all pharmacies can provide HIV prevention education and counseling. However, HIV testing and PrEP provision have not yet become a part of the standard scope of pharmacy practice. Nationwide, self-HIV tests can be purchased and self-administered in a pharmacy, and Clinical Laboratory Improvement Amendments–waived HIV tests can be provided where pharmacists are trained to provide counseling. Other tests recommended by the US Centers for Disease Control and Prevention for PrEP prescription, including those for sexually transmitted diseases and measurement of creatinine levels, are not available at pharmacies but can be performed at local laboratories. Only 9 states^13,14,15,16,17,18^ have passed legislation within the past 3 years allowing pharmacists to independently prescribe PrEP.^19,20^ Only 1 state (Virginia) in the US South has done so.^21^

The goal of this study was to elucidate the potential reach of pharmacy HIV prevention services. We specifically focused on the US South, which experiences the greatest HIV burden,^21^ using 2 approaches. First, we visually assessed the current burden of HIV in communities across the South with the current PrEP-prescribing locations and pharmacies in those communities. Next, we quantitatively calculated the PrEP facility to need ratios (PFNRs) in these communities based on their current PrEP-prescribing locations and HIV incidence. We also calculated facility to need ratios of the communities based on pharmacies, under the assumption that pharmacies provide HIV prevention services, and compared this with the current PrEP-prescribing location–based ratio.

## Methods

### Ethical Considerations

The Emory University Institutional Review Board deemed this study exempt from review since it did not include human participants. The study followed the Strengthening the Reporting of Observation Studies in Epidemiology (STROBE) reporting guideline.

### Data Sources

Data for this study were compiled from January 1 to December 31, 2021. We mapped local HIV risk with PrEP-prescribing locations and pharmacies in the southeastern US. States or specific counties included were jurisdictions identified as high-priority areas for the Ending the HIV Epidemic in the US (EHE) initiative.^22^ Specifically, 2 states (Kentucky and South Carolina) and 13 counties from 4 other states (Mecklenburg County, North Carolina; Cobb, DeKalb, Fulton, and Gwinnett counties, Georgia; Shelby County, Tennessee; and Broward, Duval, Hillsborough, Miami-Dade, Orange, Palm Beach, and Pinellas counties, Florida) were included in this analysis.

To obtain pharmacy data, active pharmacy locations at the state or county level were purchased from state Boards of Pharmacy. Each state Board of Pharmacy was contacted, and procedures to obtain data for each state were performed. Each pharmacy represents 1 facility, although multiple pharmacists could practice in each pharmacy as well as across pharmacies.

PrEP-prescribing locations were obtained from PrEP Locator, which is a national, publicly available database.^23,24^ PrEP Locator provides data on the name and address of PrEP-prescribing locations, including both public and private practice clinician offices willing to prescribe PrEP. Eligible facilities include clinics and multiple- and single-clinician practices and were verified by National Prevention Information Network staff. Thus, similar to the pharmacy location data, multiple clinicians could practice within 1 location as well as across locations. These data are updated at least once per year. Given that both pharmacy and PrEP-prescribing locations capture physical locations and do not account for the capacity of clinicians or pharmacists within PrEP-prescribing locations and pharmacies, respectively, we focused on the organizational presence of these facilities rather than internal capacity.

Data for 5-year HIV incidence per 100 000 persons (HIV risks) were collected at the zip code and county levels from AIDSVu for counties and states, respectively.^25^ AIDSVu is a publicly available database and mapping tool that integrates data from the US Centers for Disease Control and Prevention, local health departments, and other sources that allows users to explore the HIV epidemic at several spatial levels across the country.

### Geocoding

Choropleth maps of 5-year HIV risk were indexed to the county level for states and zip code level for counties. Shapefiles for county borders were obtained using the R package maps (R Project for Statistical Computing). Shapefiles of zip code borders were defined by zip code tabulation areas (ZCTAs) in the US. To map pharmacy and PrEP-prescribing locations, US Census Bureau data were obtained using the R package tigris, version 1.5.^26^ Street addresses for active pharmacy and PrEP-prescribing locations were geocoded to latitude and longitude coordinates and plotted. Geocoding was performed and base-layer maps were created and scaled using the R package ggmap that uses a Google Maps application programming interface to retrieve coordinates and maps.^27^ Specifically, each pharmacy location represents 3 pharmacies and each PrEP-prescribing location corresponds to 1 PrEP-prescribing location.

### PrEP Facility to Need Ratios

The PFNRs by state were calculated as the total number of facilities (PrEP-prescribing locations or pharmacies) divided by 5-year HIV risk per 100 000 persons. Lower PFNRs indicated lower geographic availability of locations to meet the needs of the population at risk for HIV.

### Statistical Analysis

The PFNRs for current PrEP-prescribing locations vs pharmacy locations were compared by calculating the fold-change difference between the 2 estimates. Higher values indicated greater potential effect of expansion of HIV prevention services to pharmacies. All analyses were performed using R statistical software, version 4.1.2 (R Project for Statistical Computing).^28^

## Results

**Figure 1.**
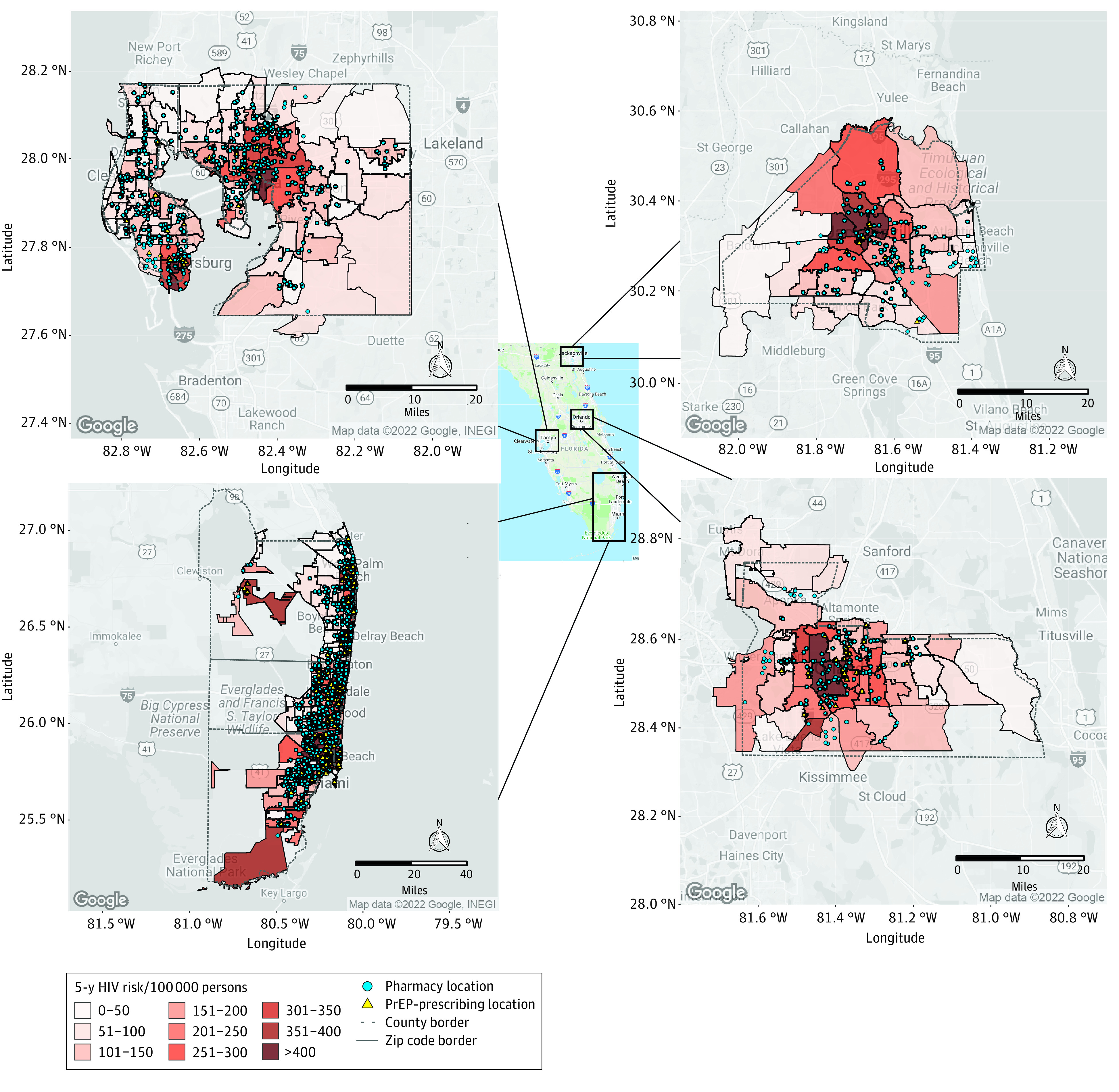
Five-Year HIV Risk by Zip Code and Pharmacy and Preexposure Prophylaxis (PrEP)–Prescribing Locations in Florida Counties Includes Broward, Duval, Hillsborough, Miami-Dade, Orange, Palm Beach, and Pinellas counties.

The spatial distribution of PrEP-prescribing locations, pharmacies, and 5-year HIV risk by ZCTA or county for high-priority areas is shown in Figures 1 through 4 and eFigures 1 and 2 in Supplement 1. The mean PFNR across all states for current PrEP-prescribing locations was 0.008 (median, 0.000 [IQR, 0.000-0.003]); for pharmacies, 0.7 (median, 0.3 [IQR, 0.01-0.1]), resulting in an overall fold increase of 80.9 across all regions if pharmacies were integrated into the provision of HIV prevention services.

In Florida, 5-year HIV risk ranged from 200 to greater than 400 cases per 100 000 persons in the immediate ZCTAs around Jacksonville and Tampa (Figure 1). In general, HIV risk was more dispersed in areas around Miami, Fort Lauderdale, and Orlando. There were 167 PrEP-prescribing locations and 2767 pharmacies across these counties. Although there were PrEP-prescribing locations in areas with high risk, there were several ZCTAs with HIV incidence greater than 400 cases per 100 000 persons with no PrEP-prescribing locations. In ZCTAs with low to medium risk, there were few to no PrEP-prescribing locations available. In contrast, pharmacies were evenly spread out across metropolitan areas and were most dense in Miami-Dade, Palm Beach, Broward, and Pinellas counties. Across all counties, there were 263 ZCTAs (of 365) without a PrEP-prescribing location, in contrast to 50 ZCTAs without a pharmacy. Across these regions in Florida, there would be a 27.8-fold increase in the PFNR if HIV prevention services were expanded to pharmacy locations (Table).

**Table.** PFNRs by State for PrEP Prescribers and Pharmacies^a^

State	PFNR, mean (median [IQR])		Fold increase with expansion to pharmacies
	PrEP prescribers	Pharmacies	
All states	0.008 (0.000 [0.000-0.003])	0.70 (0.30 [0.01-0.10])	80.9
Florida	0.002 (0.000 [0.000-0.002])	0.06 (0.03 [0.01-0.09])	27.8
Georgia	0.003 (0.000 [0.000-0.004])	0.06 (0.02 [0.01-0.07])	20.3
Kentucky	0.1 (0.1 [0.0-0.1])	20.1 (12.2 [8.6-20.3])	169.7
North Carolina	0.005 (0.000 [0.000-0.007])	0.10 (0.04 [0.03-0.09])	23.5
South Carolina	0.06 (0.00 [0.00-0.06])	5.8 (4.3 [1.4-9.0])	98.3
Tennessee	0.003 (0.000 [0.000-0.004])	0.08 (0.03 [0.01-0.08])	33.1

Abbreviations: PFNR, PrEP facility to need ratio; PrEP, preexposure prophylaxis.

^a^ Five-year HIV risks per 100 000 persons were collected at the zip code level in Florida, Georgia, North Carolina, and Tennessee and were collected at the county level in Kentucky and South Carolina.

**Figure 2.**
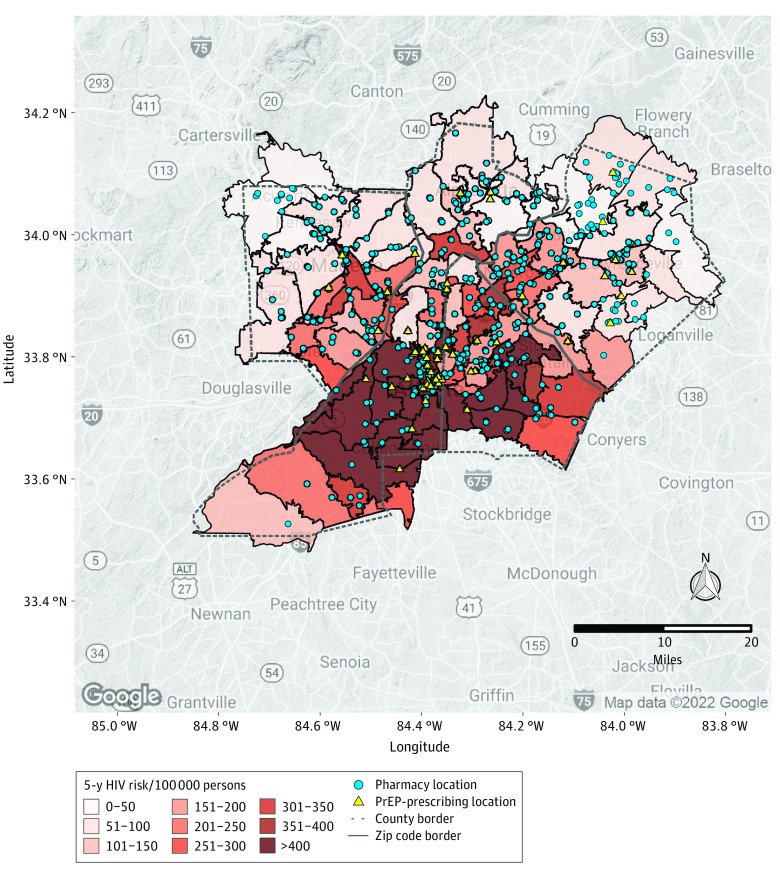
Five-Year HIV Risk by Zip Code and Pharmacy and Preexposure Prophylaxis (PrEP)–Prescribing Locations in Georgia Counties Includes Cobb, DeKalb, Fulton, and Gwinnett counties.

In Georgia, areas with the greatest 5-year HIV-risk were located in Fulton and DeKalb counties, with most ZCTAs having greater than 300 cases per 100 000 persons (Figure 2). PrEP-prescribing locations were mainly located in the downtown Atlanta area and in the northern regions of Cobb, Fulton, and Gwinnett counties. A total of 54 PrEP-prescribing locations and 781 pharmacies were found across the 4 Georgia counties. There were few to no PrEP-prescribing locations in many ZCTAs, with the highest risk in the southern areas of Fulton and DeKalb counties. Pharmacy locations were more evenly spread across the counties, although they were less dense in southern regions of Fulton and DeKalb counties. There were 59 ZCTAs (of 97) without a PrEP-prescribing location, in contrast to 6 ZCTAs without a pharmacy. In the 4 Georgia counties, the PFNR would increase by 20.3-fold if PrEP prevention services were offered in pharmacy locations.

**Figure 3.**
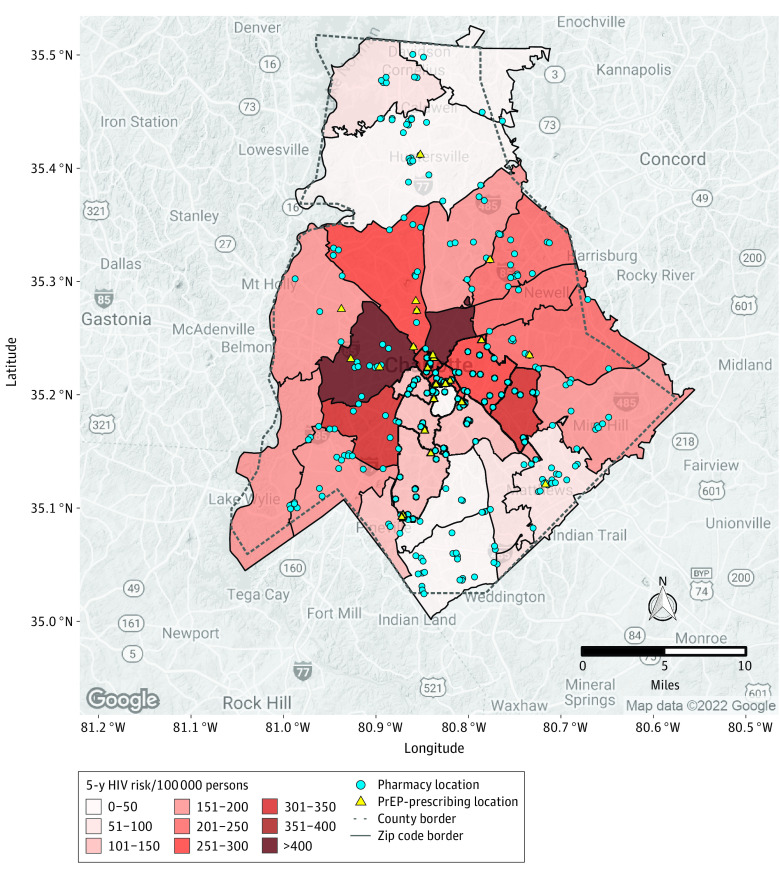
Five-Year HIV Risk by Zip Code and Pharmacy and Preexposure Prophylaxis (PrEP)–Prescribing Locations in Mecklenburg County, North Carolina

Mecklenburg County, North Carolina, had 2 ZCTAs at the highest 5-year HIV risk, with greater than 400 cases per 100 000 persons (Figure 3). Much of the region around Charlotte had more dispersed HIV risks of around 150 to 300 cases per 100 000 persons. There were 24 PrEP-prescribing locations in the county, mainly around the city of Charlotte, with few to none in the outlying areas of the county. In contrast, there were 287 pharmacies located across the county. There were only 2 ZCTAs (of 32) that did not have a pharmacy, while there were 19 without a PrEP-prescribing location. The PFNR would increase by 23.5-fold if pharmacies offered HIV prevention services in Mecklenburg County.

**Figure 4.**
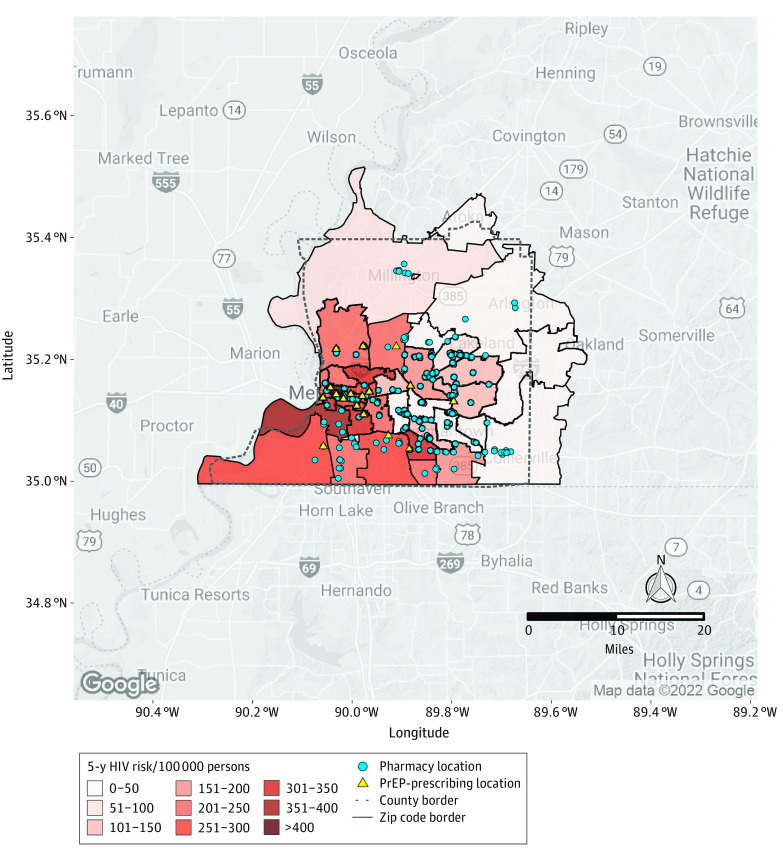
Five-Year HIV Risk by Zip Code and Pharmacy and Preexposure Prophylaxis (PrEP)–Prescribing Locations in Shelby County, Tennessee

In Shelby County, Tennessee, 5-year HIV risks were highest closer to the Memphis city center, with much lower risks in the outlying regions (Figure 4). Risks mainly ranged from 200 to 350 cases per 100 000 persons. There were 18 PrEP-prescribing locations in the county across 12 ZCTAs (of 34). A total of 208 pharmacies were found in all but 4 ZCTAs. Expanding HIV prevention services to pharmacy locations would increase the PFNR by 33.1-fold in Shelby County.

Across South Carolina, 5-year HIV risks by county ranged from 0 to greater than 40 cases per 100 000 persons, with the highest risks observed in Orangeburg and Union counties (eFigure 1 in Supplement 1). PrEP-prescribing locations were clustered around the cities of Columbia, Charleston, Myrtle Beach, and Greenville. There were a total of 54 PrEP-prescribing locations across 18 counties, resulting in 28 counties having no PrEP-prescribing location. Every county in South Carolina had an active pharmacy location. Across the state, expanding HIV prevention services to pharmacies could increase the PFNR by 98.3-fold.

The 5-year HIV risks by county for Kentucky are shown in eFigure 2 in Supplement 1. Most counties had not released their data to AIDSVu and thus HIV risks could not be estimated. PrEP-prescribing locations were mainly clustered in the Louisville and Covington metro areas. There were a total of 29 PrEP-prescribing locations in the state across 15 counties, resulting in 105 counties without a PrEP-prescribing location. There was 1 county without an active pharmacy location (Robertson); however, all other counties had at least 1 pharmacy. The PNFR (calculated only for counties with a risk estimate) would increase by 169.7-fold with the expansion of HIV prevention services to pharmacies.

## Discussion

In this study, we combined data on HIV risk operationalized as HIV incidence, PrEP-prescribing locations, and pharmacy locations to explore the potential reach of HIV prevention services offered if PrEP was available in pharmacies. We observed that PrEP-prescribing locations were unequally distributed across EHE areas in the southeastern US, with substantially fewer in areas at high risk for HIV. Most PrEP-prescribing locations were found in the urban centers of the regions presented herein, but there were many large spatial areas with high HIV risks that did not have any PrEP-prescribing locations, most prominently in Florida and Georgia in both urban and nonurban areas. In contrast, pharmacies were evenly dispersed regardless of HIV risk. Notably, in every region examined herein, more than half of all ZCTAs and counties did not have a PrEP-prescribing location, compared with at most 14% of all ZCTAs and counties that did not have a pharmacy. The mean PFNR across all states for current PrEP-prescribing locations was 0.008 and for all pharmacies was 0.7, resulting in an overall fold increase of 80.9 across all regions if pharmacies were integrated into the provision of HIV prevention services. The PFNRs were at least 20.3 times higher for pharmacies compared with PrEP-prescribing locations, indicating a substantial potential increase in HIV prevention and care services if services were expanded to pharmacies. States with the greatest potential increase in PFNRs with expansion to pharmacies included Kentucky, South Carolina, and Tennessee.

While tremendous increases in the number of PrEP-prescribing locations have occurred between 2014 and 2019, our findings and the findings of other studies^29^ suggest that the US South remains understaffed with clinicians who can offer PrEP. Specifically, the ratio of PrEP-prescribing clinicians—including physicians, nurse practitioners, and physician assistants—for every 100 persons who should receive PrEP is only 4.4 in the South compared with 8.5 in the Northeast.^29^ When surveyed, most physicians report willingness and comfort prescribing PrEP,^30,31,32^ yet PrEP uptake remains low and HIV transmission remains high. Thus, there remains a critical disconnect in the existing provision of HIV prevention services that has not reached the populations at the highest need.

Critical barriers to PrEP access, particularly in patients’ interactions with physicians, may be lack of comfort discussing sexual behaviors and requesting PrEP for fear of denial of the mediation due to its perception as specialty care by many physicians.^33^ However, data show that individuals who live in areas with more PrEP-prescribing locations are 16% more willing to use PrEP.^34^ This indicates that increased accessibility may improve acceptability of PrEP and may increase uptake as well. Therefore, pharmacies may be a strong solution to increasing access to services—including HIV prevention education and counseling as well as HIV testing and PrEP screening and dissemination—in states where legislation allows this or if alternative methods are used.^13,14,15,16,17,18,35,36^ Patients report high levels of comfort with pharmacists prescribing PrEP, particularly when they have previously obtained medication management and/or a vaccine from a pharmacist.^37^ Thus, enhancing HIV prevention care, particularly in pharmacies in areas with high HIV burden, has the potential to reduce the HIV risk of individuals in those communities.

Importantly, while our analysis makes inferences about PrEP accessibility based on location, having a PrEP-prescribing clinician in a given neighborhood does not necessarily translate to PrEP access. Individuals may have other critical barriers, including but not limited to actual risk, risk perception, transportation access, insurance coverage, and clinician preference. Moreover, our data show that to access PrEP, many individuals, particularly in South Carolina, would have to travel across multiple counties, highlighting a desert of PrEP access. In these instances especially, expanding PrEP access to pharmacies could have a considerable effect.

While the PFNR characterizes the available PrEP-prescribing locations based on the HIV incidence of the area, it is limited in that it does not inform how many PrEP-prescribing locations are needed to appropriately serve each area. In essence, this measure captures whether there is a location available for individuals who want to access PrEP and the density of these locations. Additional considerations could be taken into account in future analyses. For example, neither patient capacity nor number of physicians at PrEP-prescribing locations is known. Therefore, while some locations may be farther apart, these locations may still have the bandwidth to serve additional patients, and thus, in-house interventions that harness mobile sites could instead improve their accessibility. Regional comparisons (eg, North vs South) where the HIV incidence is declining could also be used to gauge the number of locations needed to meet the needs of the population.

### Limitations

This study has several important limitations. First, given the lack of granularity of data from some states, the availability of HIV prevention access expansion was calculated in counties for some areas and in ZCTAs for others. This may limit our ability to compare across all spatial areas and should be considered in the interpretation of the data. Moreover, in Kentucky, most counties did not provide HIV risk data; therefore, our estimates of HIV risk may be biased, and it is unclear whether these estimates are inflated or deflated.

To our knowledge, this study is the most thorough examination to date of pharmacy locations with respect to HIV. Future studies may consider multiple sources of pharmacy data. We obtained pharmacy locations from state Boards of Pharmacy to ensure pharmacies had proper licensing. However, all state Boards of Pharmacy do not provide this information. Moreover, when registering with the Boards of Pharmacy, some corporate and chain locations use their headquarters addresses, which are not necessarily located in the areas we assessed. Thus, the number of pharmacy locations and their respective PFNRs are underestimated because these pharmacies were excluded. This would suggest that the reach of pharmacies could be even greater if we included local pharmacy addresses from business data. Conversely, this analysis does not account for tele-PrEP services that may be expanding in the future as an important source for PrEP. In that case, our PrEP-prescribing locations and respective PFNRs may be overestimated.

## Conclusions

The PFNR provides critical information about structural access barriers to PrEP for individuals in the southeastern US. This has important implications for state policies that are exploring ways to increase PrEP access and uptake and thereby reduce HIV transmission in their areas. As new formulations of PrEP, such as long-acting injectable PrEP, are coming onto the market, pharmacies may also be considered an important source for interim injections once a patient has been evaluated and initiated treatment prescribed by a clinician. Expanding HIV prevention services to pharmacies in EHE areas in the southeastern US could significantly increase capacity to reach individuals who are at elevated risk for HIV transmission. Legislation aimed at allowing pharmacists to prescribe PrEP and provide HIV prevention services is a crucial next step in ending the HIV epidemic.

## Notes

Supplement 1. **eFigure 1.** Five-Year HIV Risk by County and Pharmacy and Preexposure Prophylaxis–Prescribing Locations in South Carolina **eFigure 2.** Five-Year HIV Risk by County and Pharmacy and Preexposure Prophylaxis–Prescribing Locations in Kentucky

Supplement 2.
Data Sharing Statement
