# Supplementary material for: Examination of HIV Preexposure Prophylaxis Need, Availability, and Potential Pharmacy Integration in the Southeastern US
Source: JAMA Netw Open. 2023 Jul 27;6(7):e2326028. doi: 10.1001/jamanetworkopen.2023.26028 (PMC10375311; doi:10.1001/jamanetworkopen.2023.26028)
Supplement: Supplement 1. — eFigure 1. Five-Year HIV Risk by County and Pharmacy and Preexposure Prophylaxis–Prescribing Locations in South Carolina eFigure 2. Five-Year HIV Risk by County and Pharmacy and Preexposure Prophylaxis–Prescribing Locations in Kentucky [file jamanetwopen-e2326028-s001.pdf]

Supplementary Online Content

Harrington KRV, Chandra C, Alohan DI, et al. Examination of HIV preexposure prophylaxis need, availability, and potential pharmacy integration in the southeastern US. *JAMA Netw Open*. 2023;6(7):e2326028.
doi:10.1001/jamanetworkopen.2023.26028

eFigure 1. Five-Year HIV Risk by County and Pharmacy and Preexposure Prophylaxis–Prescribing Locations in South Carolina
eFigure 2. Five-Year HIV Risk by County and Pharmacy and Preexposure Prophylaxis–Prescribing Locations in Kentucky

This supplementary material has been provided by the authors to give readers additional information about their work.

© 2023 Harrington KRV et al. *JAMA Network Open*.

eFigure 1. Five-Year HIV Risk by County and Pharmacy and Preexposure Prophylaxis–Prescribing Locations in South Carolina

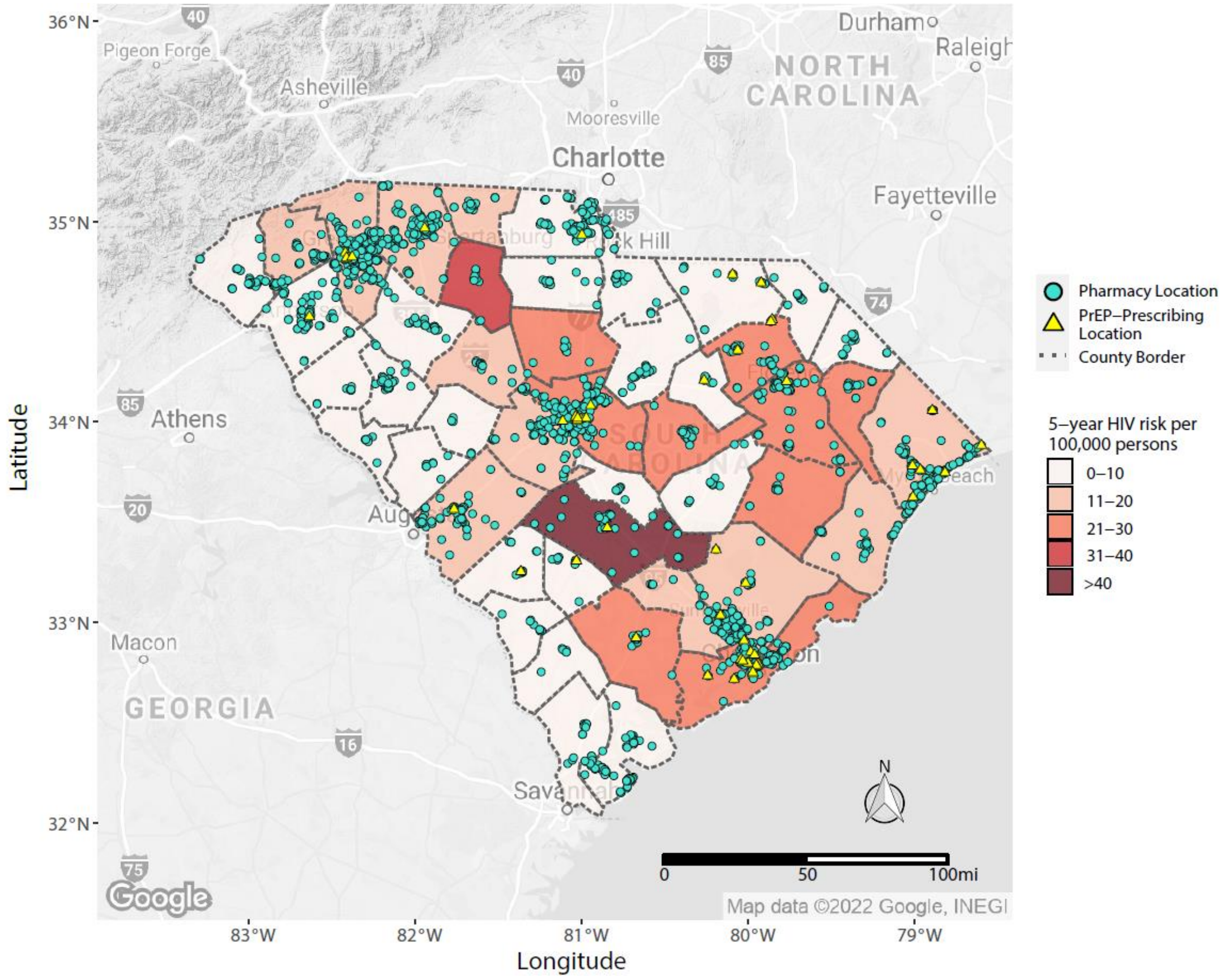

© 2023 Harrington KRV et al. *JAMA Network Open*.

eFigure 2. Five-Year HIV Risk by County and Pharmacy and Preexposure Prophylaxis–Prescribing Locations in Kentucky

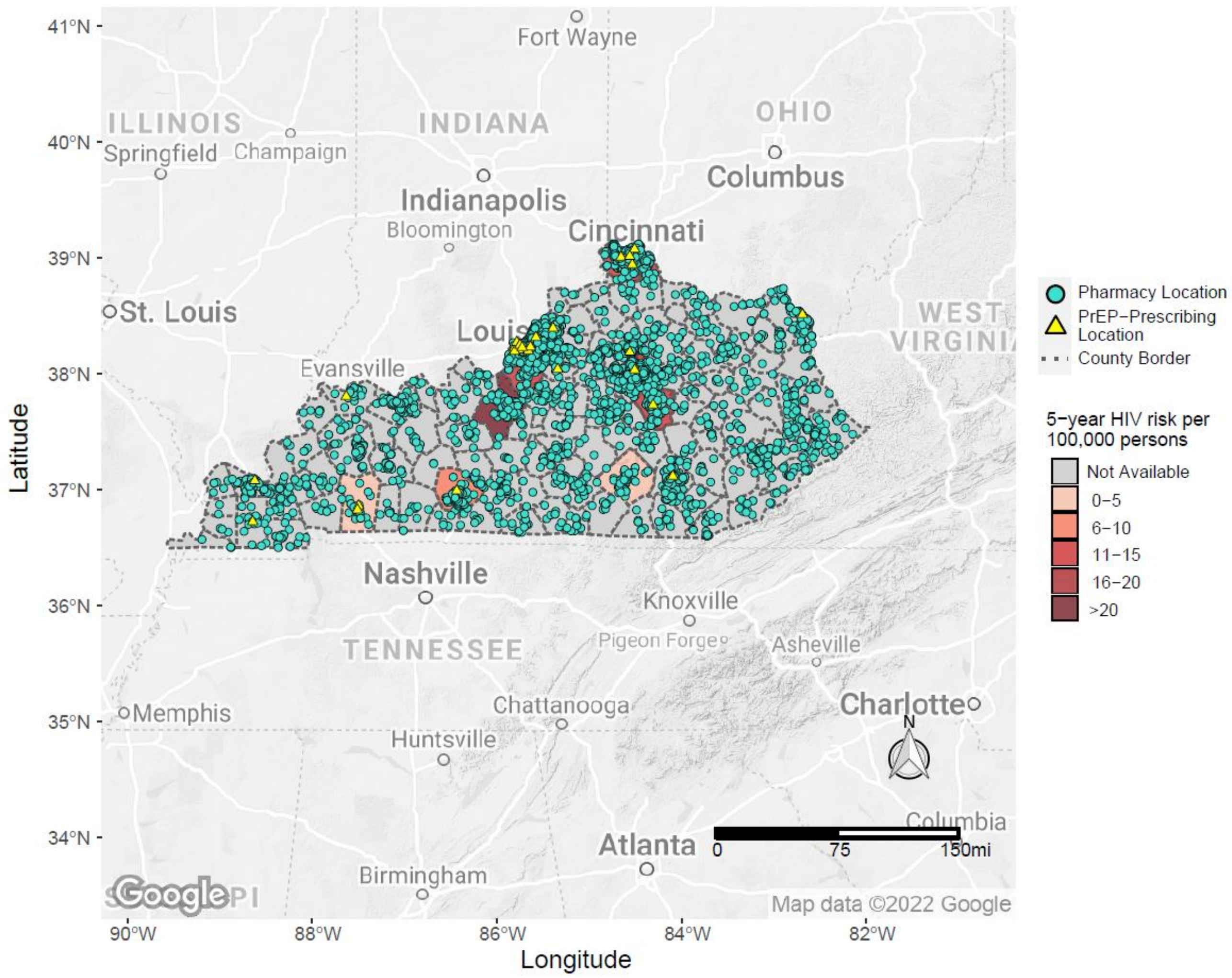

Counties with an unavailable 5-year HIV risk had not released their data to AIDSVu.

© 2023 Harrington KRV et al. *JAMA Network Open*.
