# Supplementary material for: Examination of HIV Preexposure Prophylaxis Need, Availability, and Potential Pharmacy Integration in the Southeastern US
Source: JAMA Netw Open. 2023 Jul 27;6(7):e2326028. doi: 10.1001/jamanetworkopen.2023.26028 (PMC10375311; doi:10.1001/jamanetworkopen.2023.26028)
Supplement: Supplement 2. — Data Sharing Statement [file jamanetwopen-e2326028-s002.pdf]

# Data Sharing Statement

Harrington. Examination of HIV Preexposure Prophylaxis Need, Availability, and Potential Pharmacy Integration in the Southeastern US. *JAMA Netw Open*. Published July 27, 2023. doi:10.1001/jamanetworkopen.2023.26028

**Data**
**Data available:** No

**Additional Information**
**Explanation for why data not available:** Data on PrEP-prescribing locations and 5-year HIV risk is publicly available. Data on pharmacy locations was purchased via state Boards of Pharmacy for the sole purposes of this study.
